# Circulating Cytokine Profiles and Their Relationships with Autoantibodies, Acute Phase Reactants, and Disease Activity in Patients with Rheumatoid Arthritis

**DOI:** 10.1155/2010/158514

**Published:** 2011-03-02

**Authors:** Pieter W. A. Meyer, Bridget Hodkinson, Mahmood Ally, Eustasius Musenge, Ahmed A. Wadee, Heidi Fickl, Mohammed Tikly, Ronald Anderson

**Affiliations:** ^1^Medical Research Council Unit for Inflammation and Immunity, Department of Immunology, Faculty of Health Sciences, University of Pretoria and National Health Laboratory Service-Tshwane Academic Division, P.O. Box 2034, Pretoria 0001, South Africa; ^2^Division of Rheumatology, Faculty of Health Sciences, Chris Hani Baragwanath Hospital and University of the Witwatersrand, Johannesburg 2193, South Africa; ^3^Department Internal Medicine, Faculty of Health Sciences, University of Pretoria, Pretoria 0001, South Africa; ^4^Epidemiology Centre, School of Public Health, University of the Witwatersrand, Johannesburg 2193, South Africa; ^5^Department of Immunology, School of Pathology, Faculty of Health Sciences, University of the Witwatersrand and the National Health Laboratory Services, Braamfontein, Johannesburg 2000, South Africa

## Abstract

Our objective was to analyse the relationship between circulating cytokines, autoantibodies, acute phase reactants, and disease activity in DMARDs-naïve rheumatoid arthritis (RA) patients (*n* = 140). All cytokines were significantly higher in the RA cohort than in healthy controls. Moderate-to-strong positive intercorrelations were observed between Th1/Th2/macrophage/fibroblast-derived cytokines. RF correlated significantly with IL-1*β*, IL-2, IL-4, IL-10, IL-12, G-CSF, GM-CSF, IFN-*γ*, and TNF (*P* < .0001), and aCCP and aMCV with IL-1*β*, IL-2, IL-4, and IL-10 (*P* < .0002), while IL-6 correlated best with the acute phase reactants, CRP, and SAA (*P* < .0001). In patients with a DAS28 score of ≥5.1, IFN-*γ*, IL-1*β*, IL-1Ra, TNF, GM-CSF, and VEGF were significantly correlated (*P* < .04–.001) with high disease activity (HDA). Circulating cytokines in RA reflect a multifaceted increase in immune reactivity encompassing Th1 and Th2 cells, monocytes/macrophages, and synovial fibroblasts, underscored by strong correlations between these cytokines, as well as their relationships with RF, aCCP, and aMCV, with some cytokines showing promise as biomarkers of HDA.

## 1. Introduction

The triggering events in rheumatoid arthritis (RA), especially the identity of the primary autoantigen(s), await conclusive characterization. Nonetheless, it is well recognized that ongoing inflammation of the peripheral joints with accompanying tissue damage involves complex, cytokine-driven interactions between resident synovial and infiltrating inflammatory cells [1–3], particularly T-helper 1 (Th1) cells of the effector-memory phenotype [4–6]. Recent studies have suggested that chronic inflammation in the rheumatoid joint may result from the sustained activation/dysregulation of inflammatory cytokine networks which operate independently of triggering autoantigens and T-cell receptor (TCR) ligation [7–9]. In this setting, two mechanisms, probably interactive, have been identified which appear to maintain autoantigen-independent production of inflammatory cytokines. These are (i) direct activation of a subset of effector-memory Th1 cells by cytokines signaling via the interleukin-2 receptor (IL-2R) common *γ*-chain in combination with IL-12 and IL-18, with resultant generation of interferon-*γ* (IFN-*γ*) [10, 11] and (ii) continuous activation of immune and inflammatory cells, including Th1 cells, via interaction of Toll-like receptors (TLRs) with extracellular matrix components released from damaged host tissues [12–15].

Because of the critical role of cytokine networks in perpetuating inflammatory responses in the rheumatoid joint, circulating, and/or synovial cytokines are considered to be ideal biomarkers to monitor disease onset, development and progression, either directly using cytokine multiplex immunoassays or indirectly by gene profiling approaches [16–20]. To date, however, relatively few studies have been reported in which circulating cytokine profiles have been used either as a strategy to predict disease severity and outcome in RA, or to provide insights into immunopathogenesis. Those which have been described have consistently documented significant elevations in the concentrations of a range of circulating proinflammatory cytokines [16, 21–25]. Nevertheless, these studies need to be corroborated and extended in larger groups of patients not only to establish circulating cytokine profiles in RA, but also to identify inter-relationships between cytokines of different cellular origins and their associations with conventional biomarkers and clinical markers of disease activity, and their predictive potential. 

In the current study, we have measured the concentrations of a range of circulating anti-inflammatory and proinflammatory cytokines in a relatively large cohort (*n* = 140) of predominantly African patients with early, disease modifying antirheumatic drug-naïve RA. Vascular endothelial growth factor (VEGF), which is produced by both macrophages and fibroblasts [26, 27], and which appears to correlate with disease activity and progression [28, 29], was included in the multiplex analysis.

## 2. Patients and Methods

### 2.1. Patients

A cohort of 140 patients, who met the 1987 American College of Rheumatology criteria for RA [30], were disease-modifying antirheumatic drug (DMARD) naïve, had disease duration of ≤2 years, and seen at two tertiary hospitals in South Africa, were studied. All patients were HIV-negative and receiving treatment with either diclofenac (majority) or naproxen at the time of recruitment. The 28-joint disease activity score-CRP 28 (DAS28-CRP) [31, 32] and modified Health Questionnaire Disability Index (HAQ-DI) [33] were documented. Clinically, high disease activity (HDA) was defined as DAS28 ≥ 5.1 (*n* = 63) and moderate disease activity (MDA) as a DAS28 ≥ 3.2 – < 5.1 (*n* = 62) [34]. Erosive disease was defined as the presence of marginal joint erosions on plain X-rays of the hands and feet. The study was approved by the Research Ethics Committees of the Faculties of Health Sciences of the University of Pretoria and University of the Witwatersrand.

### 2.2. Laboratory Methods

Venous blood (30 mL) was collected in endotoxin-free, silicone-coated vacutainers containing a gel separator. The blood samples were allowed to stand at room temperature to coagulate (<1 hour) followed by centrifugation (3000 rpm for 10 minutes) after which the serum was removed, aliquoted, and stored at minus 20°C until performance of the various assays described below.

#### 2.2.1. Autoantibodies and Acute Phase Reactants

Rheumatoid factor (composite IgM, IgG, IgA), CRP, and SAA were assayed by nephelometry (Siemens Healthcare Diagnostics, BN Prospec Nephelometer, Newark, USA). As per supplier controls, RF, CRP, and SAA results were considered positive when the concentrations were greater than 11 IU/mL, 5 *μ*g/mL and 6.8 *μ*g/mL, respectively. Anticyclic citrullinated peptide antibodies (aCCP) were measured by an immunofluorometric procedure using the Immunocap 250 system and reagents and controls provided by the manufacturer (Phadia AB, Uppsala, Sweden), and a concentration >10 U/mL was deemed positive. Antimodified citrullinated vimentin antibodies (aMCV) were measured using an ELISA assay (Orgentec Diagnostika GmbH, Mainz, Germany), and a concentration >20 U/mL was deemed positive.

#### 2.2.2. Serum Cytokines, Chemokines, and Growth Factors

These were measured using the Bio-Plex suspension array system (Bio-Rad Laboratories Inc, Hercules, CA, USA) which utilizes Luminex xMAP multiplex technology to enable simultaneous detection and quantitation of multiple different analytes in a single sample. The system uses an array of microspheres in liquid suspension, conjugated with a monoclonal antibody specific for a target protein. The beads contain different ratios of two spectrally distinct fluorophores, thereby assigning a unique spectral identity. These antibody-coupled, colour-coded beads were then incubated with the serum sample (1/4 dilution), washed, followed by addition of a biotinylated detection antibody, washed again, and finally incubated with streptavidin-phycoerythrin. A wide range of standards (0.38–91756.00 pg/mL) was used to enable quantitation of the individual cytokines using a Bio-Plex array reader with a dual laser detector and real-time digital signal processing. The following analytes were measured simultaneously using a 17-plex test kit: IL-1*β*, IL-1Ra, Il-2, IL-4, IL-6, IL-7, IL-8, IL-10, IL-12, IL-17A, IFN-*γ*, TNF, G-CSF, GM-CSF, CCL2, CCL4, and VEGF, broadly representative of Th1/Th2/Th17, and macrophage/fibroblasts. The upper limit of normal for these analytes was calculated as the mean + 1SD for 10 healthy control subjects (5 female, 5 male, average age 44.4 ± 15.0 years, ranging from 26–65 years of age).

#### 2.2.3. Statistical Methods

Statistical Analysis was performed using STATA statistical software. Correlation coefficients were derived from correlation matrices using the nonparametric Spearman's rank correlation test with Holm's method *P* value correction for multiple testing. A *P* value <  .05 was considered as significant.

## 3. Results

### 3.1. Demographic, Clinical, and Laboratory (Autoantibodies, Acute Phase Reactants) Data

These are shown in Tables 1 and 2 for the total cohort and the MDA and HDA subgroups, respectively. RF, aCCP, and aMCV were positive in 79%, 79%, and 74% of RA patients, respectively, while CRP and SAA levels were raised in 71% and 68% of patients, respectively. The mHAQ-DI did not correlate with any of the laboratory parameters or DAS28, while the DAS28 score correlated with CRP and SAA (*r* = 0.52 and *r* = 0.42, resp., *P* < .0001 for both), in keeping with Emery et al. [35] Not surprisingly, aCCP and aMCV were strongly correlated (*r* = 0.76, *P* < .0001) as were CRP and SAA (*r* = 0.82, *P* < .0001), while only weak correlations were detected between these autoantibodies and the acute phase reactants. RF correlated moderately with aCCP and aMCV (*r* = 0.51, *P* = .0002 and *r* = 0.44, *P* = .0014, resp.), and weakly with CRP and SAA (*r* = 0.2, *P* < .02 for both).

### 3.2. Circulating Cytokines, Chemokines, and Growth Factors in Healthy Controls and RA Patients

With the exception of TNF, IL-12, G-GSF, and VEGF (mean values of 21 ± 3, 34 ± 37, 34 ± 16, and 176 ± 189 pg/mL, resp.), the mean circulating concentrations of all the other cytokines ranged from 0–6 pg/mL in the group of healthy subjects with no meaningful differences between males and females. The results for the total cohort of RA patients, as well as for the MDA and HDA subgroups are shown in Tables 3 and 4, respectively. The serum concentrations of the cytokines, chemokines and growth factors were significantly elevated in the total RA cohort, relative to those of the healthy control subjects, and those previously reported for healthy adult humans [36], although the spread of values for each cytokine was considerable. The only exception was IL17A, for which only very modest elevations were evident. No significant differences were observed between the MDA and HDA groups.

### 3.3. Correlations between Cytokines/Chemokines/Growth Factors

These results are shown in Table 5 with strong correlations highlighted in bold type and moderate and weak correlations in plain type. Moderate-to-strong correlations between cytokines of Th1 cell (IL-2, IFN-*γ*, GM-CSF), Th2 cell (IL-4, IL-6, IL-10), monocyte/macrophage (IL-1*β*, IL-6, IL-12, TNF, G-CSF), and fibroblast (IL-7) origin were observed in the total cohort of RA patients. Of the chemokines, only CCL2 was moderately correlated with the Th1/Th2/macrophage/fibroblast cytokines (*r* = 0.46–0.66, *P* ≤ .0001–.0007). VEGF, perhaps not surprisingly, given its probable fibroblast and macrophage origins [26, 27], correlated best with IL-7 (*r* = 0.55, *P* < .0001) and IL-12 (*r* = 0.45, *P* = .0009), but only weakly or not at all with other cytokines.

### 3.4. Correlations with RF, aCCP, and aMCV

These results are shown in Table 6 for the total cohort of RA patients. RF correlated moderately with IL-1*β*, IL-4, IL-7, IL-12, G-CSF, GM-CSF, IFN-*γ*, and TNF and weakly-to-moderately with IL-Ra, IL-2, IL-6, IL-8, IL-10, IL17A, VEGF, CCL2, and CCL4. Similar, albeit somewhat weaker, correlations were observed between these same cytokines and aCCP and aMCV antibodies. In the case of the acute phase reactants, IL-6, not surprisingly, was found to correlate moderately with CRP and SAA (*r* = 0.49 and 0.52, resp., *P* < .0001 for both), while weak or no correlations were noted with the other cytokines (not shown). 

Interestingly, VEGF which correlated only weakly with the Th1/Th2/macrophage/fibroblast cytokines in the total cohort demonstrated considerably stronger correlations with these cytokines in the HDA subgroup. In this subgroup, but not in the total cohort, strong and significant correlations of VEGF with RF, aCCP, and aMCV were also evident as well as a weak correlation with X-ray (Larsen) scores as shown in Table 7.

### 3.5. Associations of the DAS28 Clinical Scores in the Total Cohort of Patients and the HDA and MDA Subgroups with Circulating Biomarkers of Disease Activity

No significant relationships were evident between the DAS28 scores, disease activity and any of the serum cytokines, chemokines, and growth factors in the total RA cohort or the MDA subgroup (data not shown). In the HDA subgroup, however, DAS28 scores, but not disease duration, showed significant correlations with serum IFN-*γ* (*r* = 0.49, *P* = .0160), IL-1*β* (*r* = 0.52, *P* = .0088), IL-1Ra (*r* = 0.62, *P* = .0013), TNF (*r* = 0.58, *P* = .0033), and GM-CSF (*r* = 0.58, *P* = .0033).

## 4. Discussion

Multiplex analysis of circulating cytokines in RA has the potential not only to provide novel insights into the immunopathogenesis of RA, but also to identify subgroups of patients at risk for development of severe disease. To date, however, this important topic has been addressed in a limited number of studies [16, 19, 20, 22–25, 37]. These have shown promise, particularly two recent studies which have described generalised elevations in circulating cytokines, especially Th1/Th2/macrophage cytokines, which precede the onset of clinical disease and are also markers of disease severity [20, 37, 38]. However, there is clearly a need for corroboration and extension in larger groups of patients. In the current study, we have investigated circulating cytokine profiles, representative of Th1, Th2, and Th17 cells, monocytes/macrophages, and fibroblasts, in a large group of DMARD-naïve RA patients of disease duration <2 years. Notwithstanding characterization of circulating cytokine profiles, the major objectives of the current study were to identify inter-relationships between cytokines, as well as correlations with RA-associated autoantibodies, acute phase reactants, and clinical disease activity. These were analysed in the total cohort of patients, as well as in the MDA and HDA subgroups. 

In the case of the total cohort, and in keeping with previous studies [16, 19, 20, 22–25, 37], we observed a general increase in the circulating levels of all of the anti-inflammatory/proinflammatory cytokines, growth factors and chemokines. IL17A was the exception, being barely detectable and only modestly intercorrelated with the other cytokines. In contrast, cytokines primarily of Th1 and Th2 lymphocyte origin, as well as those derived mainly from monocytes/macrophages and fibroblasts, were not only elevated, but also highly intercorrelated in the total cohort, and strongest in the HDA subgroup. The strong correlations of the Th2 cytokines, IL-4, and IL-10 [39], with those of the proinflammatory cytokines, IL-1*β*, IL-2, IL-6, IL-7, IL-12, G-CSF, GM-CSF, IFN-*γ*, and TNF, probably reflect the efforts of these cells to suppress the reactivity of their Th1 counterparts, and to redirect the phenotype and functions of classically IFN-*γ*-activated M1 macrophages [40, 41]. It is, however, noteworthy that IL-10 also originates from cell types other than Th2 cells, and M2-derived IL-10 is also likely to fulfill an anti-inflammatory function underscoring the plasticity of transitions between various cell phenotypes according to the cytokine environment. Notwithstanding its anti-inflammatory activity, IL-4 may also play a proinflammatory role in RA by driving the activity of autoreactive B cells, possibly underscored by the associations of IL-4 with aCCP and aMCV. In addition to effects on Th1 cells and M1 macrophages, the Th2 cytokines IL-4 and IL-10 also suppress the proliferation and proinflammatory activities of fibroblasts [42–45]. Importantly, the consistency and magnitude of the correlations between the cytokines suggest that these are unlikely to be spurious, and also attest to the robustness of the multiplex cytokine assay system. Although our group of healthy control subjects was relatively small (*n* = 10), their low levels of circulating cytokines are comparable with those reported from a much larger series of healthy subjects [36].

In keeping with the increasing recognition of the involvement of IL-7 in orchestrating chronic inflammation in the rheumatoid joint [46–50], this cytokine was found to be highly correlated with IL-12 in particular, as well as with IFN-*γ*. Synovial fibroblasts, activated by IL-1*β* and TNF, as well as macrophages, are likely to be the major source of IL-7. Interestingly, the predominant lymphocyte subset in the rheumatoid joint has been reported to be of the Th1 effector-memory phenotype, expressing functional IL-12R, IL-18 R*α*, and CCR5 [11]. These cells are directly activated to secrete IFN-*γ* by cytokines such as IL-2, IL-7, and IL-15, but not IL-4, which signal via the IL-2R common *γ*-chain in combination with IL-12 and IL-18, independently of TCR ligation [11]. Although of considerable interest, further studies are necessary to confirm the role of this autoantigen-independent mechanism of perpetuation of inflammation in RA.

Circulating IFN-*γ* was strongly correlated with the predominantly macrophage-derived cytokines IL-1*β*, IL-12, TNF, and G-CSF. This is in keeping with the classically, IFN-*γ*-activated macrophage of the M1 phenotype being a major source of proinflammatory cytokines in the inflamed RA joint [51]. Of the 3 chemokines measured, IL-8, CCL2, and CCL4, only CCL2 showed moderate correlations with the various Th1/Th2, macrophage and fibroblast cytokines, in the total cohort, possibly reflecting the predominantly macrophage/fibroblast origins of this monocyte-targeted chemokine.

Circulating VEGF, which has been reported to predict disease severity and progression in RA [28, 29, 52], correlated poorly with the other cytokines with the exceptions of IL-7 in particular, and IL-12, possibly reflecting the fibroblast and macrophage origins, respectively, of VEGF [26, 27]. However, when the total cohort of RA patients was subdivided into those with MDA and HDA, significant correlations were observed between VEGF and the Th1/Th2/macrophage/fibroblast cytokines, as well as with RF, aCCP, and aMCV in the HDA group, substantiating the role of VEGF as a predictor of severity of disease in our cohort.

With respect to correlations of the Th1, Th2, Th17, monocyte/macrophage, and fibroblast cytokines, with traditional biomarkers of disease activity in the total cohort, RF correlated moderately with IL-1*β*, IL-4, IL-7, IL-12, G-CSF, GM-CSF, IFN-*γ*, and TNF, with weaker, but nonetheless significant correlations detected between these same cytokines, and aCCP and aMCV. Not surprisingly, the acute phase reactants, CRP and SAA, correlated best with IL-6, while weak correlations were evident with most of the other cytokines. Given the strong association of RF, aCCP, and aMCV with disease severity and poor prognosis in RA [53–56], these findings are compatible with the involvement of the Th1 lymphocyte/macrophage/fibroblast axis in the immunopathogenesis of RA [5, 57]. This contention is underscored by the significant correlations of IL-1*β*, IL-1Ra, TNF, IFN-*γ*, and GM-CSF with the DAS28 score observed in the HDA subgroup. Although a role for Th2 cells in disease pathogenesis cannot be excluded, the association of the Th2 cytokines, IL-4, and IL-10, with RF, aCCP, and aMCV most likely reflects their anti-inflammatory activities. A dynamic interaction between the production of Th1 and Th2 cytokines with opposing pro- and anti-inflammatory activities may regulate the rate and severity of damage to cartilage and bone in established RA. This in turn may explain the relatively weak correlations of acute phase reactants with cytokines and autoantibodies.

Transiently elevated concentrations of cytokine in synovial fluid have been reported with certain cytokines of T cell, macrophage, and stromal cell origin being elevated in early disease, but apparently undetectable in established disease [58]. The inflamed synovium is infiltrated by pathogenic effector T cells and autoreactive B cells activated by unknown triggers where they orchestrate local joint inflammation. It has been clearly demonstrated that the elevated synovial, as well as circulating cytokines, are a common manifestation of the persistent state of inflammation and are predictive of disease severity [1, 16, 20, 24, 38, 58–62]. 

The limitations of the current study include our focus on circulating, as opposed to synovial cytokines [63], as well as the recruitment of patients with established, rather than very early disease. In defence of these strategies, measurement of synovial cytokines presents logistical and ethical issues and is relatively impractical from a laboratory diagnostic perspective, while identification of patients with very early disease is particularly difficult in the health care setting of a developing country. The strengths of the study, on the other hand, include the relatively large number of patients recruited to the study, all of whom were DMARD-naïve, as well as the range of circulating markers of disease activity which were evaluated.

In conclusion, the current study has demonstrated a circulating cytokine profile in patients with established RA which appears compatible with Th1 cell/macrophage/fibroblast activation and a possible counter-regulatory role of Th2 cells. This is underscored by relatively strong correlations between cytokines and established biomarkers, especially RF, as well as aCCP and aMCV antibodies. IL-1*β*, IL-1Ra, TNF, IFN-*γ*, GM-CSF, and VEGF in particular were significantly associated with HDA and show promise as adjunctive diagnostic/prognostic biomarkers, either individually or in combination. The clinical utility of this strategy will, however, depend on the outcome of large, multicentre follow-up studies.

## Figures and Tables

**Table 1:** Demographic, laboratory, and clinical data for the group of RA patients (*n* = 140).

Parameters			Quintiles		
	Mean ± SD	Q1 (p25)	Q2 (Median)	Q3 (p75)	Range
Age (years)	48 ± 12	41	48	56	20–75
Female (%)	115 (82.1)				
Ethnic origin: Black (%)	111 (87.0)				
DAS28-CRP	5.7 ± 1.2	4.8	5.9	6.6	2.3–8.0
HDA (DAS28 ≥ 5.1) (%)	63 (45.0)				
LDA (DAS28 ≥ 3.2 & <5.1) (%)	62 (44.3)				
mHAQ-DI	3.2 ± 1.6	2.0	3.5	4.5	0.0–6.0
Disease duration (months)	12 ± 7	6	10	18	0–25
SJC	10 ± 6	4	9	14	0–27
X-ray erosions (%)	65/121 (53.7)				
RF (IU/mL)	493 ± 803	40	171	579	4–5350
aCCP (U/mL)	654 ± 621	80	490	1082	2–2431
aMCV (U/mL)	594 ± 679	104	326	775	16–3088
CRP (mg/L)	23.0 ± 2.5	5.1	13.6	30.6	0.3–164
SAA (*μ*g/mL)	61 ± 124	5	16	60	1–882

DAS28: Disease activity score for 28 joints, HDA: High disease activity, LDA: Low disease activity, mHAQ-DI: modified Health assessment questionnaire-disease Index, SJC: Swollen Joint Count, RF: Rheumatoid factor, aCCP: anticyclic citrullinated peptide antibodies, aMCV: Antimodified citrullinated vimentin antibodies, CRP: C-reactive protein, SAA: Serum amyloid A.

**Table 2:** Demographic, laboratory, and clinical data for the LDA (*n* = 62) and HDA (*n* = 63) groups of RA patients.

Parameters			MDA					HDA		
			Quintiles					Quintiles		
	Mean ± SD	Q1 (p25)	Q2 (Median)	Q3 (p75)	Range	Mean ± SD	Q1 (p25)	Q2 (Median)	Q3 (p75)	Range
Age (years)	46 ± 10	41	45	53	22–68	49 ± 12	45	50	57	20–75
Female (%)	47 (75)					50 (80)				
Ethnicity: Black (%)	48 (78)					59 (94)				
DAS28-CRP	4.5 ± 0.5	4.3	4.7	4.8	3.3–5.0	6.5 ± 0.6	6.2	6.6	6.9	5.2–8.7
mHAQ-DI	3.3 ± 1.7	2.0	3.6	4.5	0.0–6.0	1.8 ± 0.8	1.3	2.1	2.4	0.0–3.0
Disease duration (months)	13 ± 8	7	11	20	2–24	11 ± 8	5	9	18	2–24
SJC	5 ± 3	3	5	7	0–15	14 ± 6	10	13	17	4–28
X-ray erosions (%)	28/53 (53)					29/57 (51)				
RF (IU/mL)	480 ± 681	57	177	559	4–2860	491 ± 812	21	178	654	10–5350
aCCP (U/mL)	618 ± 639	51	343	1001	2–2026	734 ± 667	89	599	1213	2–2527
aMCV (U/mL)	597 ± 731	90	319	710	20–3028	638 ± 727	108	334	868	16–3088
CRP (mg/L)	8 ± 10	2	5	8	1–40	36 ± 38	10	27	37	0–198
SAA (*μ*g/mL)	25 ± 48	2	5	24	1–238	91 ± 153	8	35	124	1–882

DAS28: Disease activity score for 28 joints, HDA: High disease activity, LDA: Low disease activity, mHAQ-DI: modified Health assessment questionnaire-disease Index, SJC: Swollen Joint Count, RF: Rheumatoid factor, aCCP: anticyclic citrullinated peptide antibodies, aMCV: Antimodified citrullinated vimentin antibodies, CRP: C-reactive protein, SAA: Serum amyloid A.

**Table 3:** Serum cytokine, chemokine, and growth factor values for the total RA cohort *n* = 140.

	Mean ± SD*	Q1(p25)*	Q2 (Median)*	Q3 (p75)*	Range*
IL-1*β*	24.8 ± 48	1.4	5.3	20.0	0–269
IL-Ra	363.5 ± 793	25.3	74.8	266.1	0–5012
IL-2	44.7 ± 155	0.0	0.0	30.2	0–1320
IL-4	30.5 ± 84	0.1	3.6	13.3	0–598
IL-6	64.7 ± 130	6.8	18.3	65.5	0–1078
IL-7	221.1 ± 741	4.9	22.2	89.5	0–6825
IL-8	295.5 ± 3111	5.0	9.3	20.2	0–36699
IL-10	41.6 ± 134	3.9	10.7	25.8	0–1172
IL-12	123.9 ± 404	2.2	11.0	54.3	1–3105
IL-17A	6.0 ± 26	0.0	0.0	0.26	0–229
G-CSF	226.9 ± 842	0.0	14.3	57.9	0–8764
GM-CSF	112.8 ± 375	0.0	0.0	31.0	0–3726
IFN-*γ*	580.1 ± 1588	0.0	31.3	195.5	0–10922
CCL2	89.0 ± 200	0.0	50.7	107.4	0–1771
CCL4	132.6 ± 94	70.4	108.2	171.7	27–544
TNF	149.6 ± 434	4.3	11.6	61.9	1–2952
VEGF	450.4 ± 712	59.4	166.4	518.8	0–4503

*results in pg/mL.

**Table 4:** Circulating cytokine data for the MDA (*n* = 62) and HDA (*n* = 63) groups of RA patients..

	MDA					HDA				
Cytokines	Quintiles					Quintiles				
	Mean±SD	Q1 (p25)	Q2 (Median)	Q3 (p75)	Range	Mean±SD	Q1 (p25)	Q2 (Median)	Q3 (p75)	Range
IL-1*β*	19 ± 29	3	4	26	0–122	30 ± 60	1	5	17	0–269
IL-1Ra	291 ± 495	28	73	266	0–2994	429 ± 973	26	90	252	0–5012
IL-2	33 ± 88	0	0	31	0–516	33 ± 68	0	0	27	0–297
IL-4	33 ± 75	0	5	15	0–361	20 ± 0.46	0	3	11	0–254
IL-6	51 ± 79	6	16	60	0–309	73 ± 120	11	27	86	0–792
IL-7	160 ± 476	5	21	95	0–3324	282 ± 954	7	23	78	0–6852
IL-8	54 ± 167	5	9	18	0–997	612 ± 4620	8	11	23	0–36699
IL-10	35 ± 82	3	11	22	0–472	51 ± 178	6	10	27	0–1172
IL-12	172 ± 401	2	11	48	1–226	117 ± 407	3	13	64	1–3105
IL-17A	8 ± 37	0	0	0	0–229	3 ± 9	0	0	0	0–46
IFN-*γ*	585 ± 1444	3	48	350	0–7541	527 ± 1330	0	27	160	0–6431
G-CSF	181 ± 461	1	16	55	0–2285	179 ± 423	0	7	64	0–1664
GM-GSF	87 ± 211	0	0	24	0–1048	101 ± 236	0	0	41	0–978
TNF	161 ± 452	5	11	54	1–2952	131 ± 392	5	12	69	1–2665
VEGF	475 ± 769	54	139	513	0–3582	419 ± 502	70	198	534	9–2196
CCL2	69 ± 85	0	41	109	0–417	112 ± 276	14	55	103	0–1771
CCL4	117 ± 64	68	95	163	27–291	159 ± 116	89	119	199	32–643

Values in pg/mL.

**Table 5:** Correlations between the serum cytokines, chemokines, and growth factor concentrations in the total cohort of RA patients (*n* = 140).

	IL-1*β*	IL-1Ra	IL-2	IL-4	IL-6	IL-7	IL-8	IL-10	IL-12	IL-17	G-CSF	GM-CSF	IFN-*γ*
TNF	**0.85**	0.64	**0.77**	**0.86**	0.59	0.50	0.23	0.70	0.68	0.52	**0.75**	**0.79**	**0.81**
	**<.0001**	<.0001	**<.0001**	**<.0001**	<.0001	<.0001	.0062	<.0001	<.0001	<.0001	**<.0001**	**<.0001**	**<.0001**

IFN-*γ*	**0.86**	0.66	**0.80**	**0.95**	0.56	**0.73**	0.31	**0.72**	**0.79**	0.50	**0.81**	**0.82**	
	**<.0001**	<.0001	**<.0001**	**<.0001**	<.0001	**<.0001**	.0002	**<.0001**	**<.0001**	<.0001	**<.0001**	**<.0001**	

GM-CSF	**0.86**	**0.72**	**0.89**	**0.87**	0.59	0.50	0.26	**0.77**	0.67	0.48	**0.79**		
	**<.0001**	**<.0001**	**<.0001**	**<.0001**	<.0001	<.0001	.0015	**<.0001**	<.0001	<.0001	**<.0001**		

G-CSF	**0.77**	0.62	**0.71**	**0.86**	0.47	0.49	0.22	0.63	0.55	0.48			
	**<.0001**	<.0001	**<.0001**	**<.0001**	<.0001	<.0001	.0083	<.0001	<.0001	<.0001			

IL-12	**0.84**	0.53	**0.79**	**0.82**	0.45	**0.84**	0.35	**0.90**					
	**<.0001**	<.0001	**<.0001**	**<.0001**	<.0001	**<.0001**	<.0001	**<.0001**					

IL-10	**0.80**	0.68	**0.76**	**0.73**	0.69	0.63	0.23						
	**<.0001**	<.0001	**<.0001**	**<.0001**	<.0001	<.0001	.0237						

IL-7	0.61	0.34	0.61	**0.70**	0.35								
	<.0001	<.0001	<.0001	**<.0001**	<.0001								

IL-4	**0.88**	**0.70**	**0.81**										
	**<.0001**	**<.0001**	**<.0001**										

IL-2	**0.83**	**0.77**											
	**<.0001**	**<.0001**											

IL-1Ra	**0.79**												
	**<.0001**												

*For each pair of values, the correlation coefficient (*r*) is uppermost with the corresponding *P* value underneath.

Strong correlations (*r* ≥ 0.7) are highlighted in bold type, and moderate and weak correlations in plain type.

**Table 6:** Correlations of RF, aMCV, and aCCP with cytokines, chemokines, and growth factors in the total cohort of RA patients (*n* = 140).

	RF	aMCV	aCCP
IL-1*β*	0.5199	0.3823	0.3264
	0.0001	0.0061	0.0207

IL-1Ra	0.4193	0.4208	ns
	0.0024	0.0023	

IL-2	0.4737	0.4399	0.3607
	0.0005	0.0014	0.0101

IL-4	0.6718	0.5277	0.5096
	0.0000	0.0001	0.0002

IL-6	0.3970	0.4693	0.4346
	0.0043	0.0006	0.0016

IL-7	0.5021	0.3244	0.3665
	0.0002	0.0215	0.0088

IL-8	0.3407	ns	ns
	0.0155		

IL-10	0.4339	0.4540	0.3450
	0.0016	0.0009	0.0142

IL-12	0.5732	0.4526	0.4396
	0.0000	0.0010	0.0014

IL-17A	0.2910	ns	ns
	0.0403		

G-CSF	0.6254	0.5140	0.4969
	0.0000	0.0001	0.0002

GM-CSF	0.6150	0.5639	0.4552
	0.0000	0.0000	0.0009

IFN-*γ*	0.6561	0.5562	0.5473
	0.0000	0.0000	0.0000

TNF	0.5667	0.4920	0.4120
	0.0000	0.0003	0.0029

VEGF	0.4405	ns	ns
	0.0014		

CCL2	0.3624	ns	ns
	0.0097		

CCL4	0.3597	0.4444	0.3790
	0.0103	0.0012	0.0066

RF: Rheumatoid factor, aCCP: anticyclic citrullinated peptide antibodies, aMCV: Antimutated citrullinated vimentin antibodies.

ns: not significant.

**Table 7:** Correlations of VEGF with autoantibodies, circulating cytokines, and X-ray scores in the total cohort (*n* = 140) and HDA group (*n* = 63).

	Total	HDA
	*r*(*p*)	*r*(*p*)
RF	0.44 (0.0014)	0.60 (0.0023)
SAA	ns	ns
CRP	ns	ns
aMCV	ns	0.59 (0.0023)
aCCP	ns	0.67 (0.0003)
IL-1*β*	0.29 (0.0433)	0.48 (0.0190)
IL-1Ra	ns	0.41 (0.0474)
IL-2	0.40 (0.0038)	0.61 (0.0014)
IL-4	0.36 (0.0098)	0.65 (0.0006)
IL-7	0.55 (<0.0000)	0.65 (0.0006)
IL-8	ns	0.41 (0.0448)
IL-10	0.38 (0.0072)	0.46 (0.0246)
IL-12	0.46 (0.0009)	0.60 (0.0021)
G-CSF	0.31 (0.0306)	0.55 (0.0052)
GM-CSF	0.37 (0.0086)	0.69 (0.0002)
IFN-*γ*	0.37 (0.0082)	0.66 (0.0004)
TNF	ns	0.53 (0.0078)
CCL2	0.40 (0.0044)	ns
CCL4	ns	0.56 (0.0044)
Larsen	ns	0.42 (0.0409)

RF: Rheumatoid factor, SAA: Serum amyloid A, CRP: C-reactive protein, aCCP: anticyclic citrullinated peptide antibodies, aMCV: Antimodified citrullinated vimentin antibodies.

ns: not significant.

## References

[1] Choy EHS, Panayi GS (2001). Cytokine pathways and joint inflamation in rheumatoid arthritis. *New England Journal of Medicine*.

[2] Firestein GS (2003). Evolving concepts of rheumatoid arthritis. *Nature*.

[3] Klareskog L, Catrina AI, Paget S (2009). Rheumatoid arthritis. *The Lancet*.

[4] Dolhain RJEM, Van Der Heiden AN, Ter Haar NT, Breedveld FC, Miltenburg AMM (1996). Shift toward T lymphocytes with a T helper 1 cytokine-secretion profile in the joints of patients with rheumatoid arthritis. *Arthritis and Rheumatism*.

[5] Yamada H, Nakashima Y, Okazaki K (2008). Th1 but not Th17 cells predominate in the joints of patients with rheumatoid arthritis. *Annals of the Rheumatic Diseases*.

[6] Wang CR, Liu MF (2003). Regulation of CCR5 expression and MIP-1*α* production in CD4+ T cells from patients with rheumatoid arthritis. *Clinical and Experimental Immunology*.

[7] McInnes IB, Leung BP, Liew FY (2000). Cell-cell interactions in synovitis. Interactions between T lymphocytes and synovial cells. *Arthritis Research*.

[8] Firestein GS, Zvaifler NJ (2002). How important are T cells in chronic rheumatoid synovitis? II. T cell-independent mechanisms from beginning to end. *Arthritis and Rheumatism*.

[9] Müller-Ladner U, Pap T, Gay RE, Neidhart M, Gay S (2005). Mechanisms of disease: the molecular and cellular basis of joint destruction in rheumatoid arthritis. *Nature Clinical Practice*.

[10] Aita T, Yamamura M, Kawashima M (2004). Expression of interleukin 12 receptor (IL-12R) and IL-18R on CD4+ T cells from patients with rheumatoid arthritis. *Journal of Rheumatology*.

[11] Sattler A, Wagner U, Rossol M (2009). Cytokine-induced human IFN-*γ*-secreting effector-memory Th cells in chronic autoimmune inflammation. *Blood*.

[12] Imanishi T, Hara H, Suzuki S, Suzuki N, Akira S, Saito T (2007). Cutting edge: TLR2 directly triggers Th1 effector functions. *Journal of Immunology*.

[13] Brentano F, Kyburz D, Schorr O, Gay R, Gay S (2005). The role of Toll-like receptor signalling in the pathogenesis of arthritis. *Cellular Immunology*.

[14] Abdollahi-Roodsaz S, Joosten LAB, Roelofs MF (2007). Inhibition of toll-like receptor 4 breaks the inflammatory loop in autoimmune destructive arthritis. *Arthritis and Rheumatism*.

[15] Li M, Zhou Y, Feng G, Su SB (2009). The critical role of toll-like receptor signaling pathways in the induction and progression of autoimmune disease. *Current Molecular Medicine*.

[16] Khan IH, Krishnan VV, Ziman M (2009). Comparison of multiplex suspension array large-panel kits for profiling cytokines and chemokines in rheumatoid arthritis patients. *Cytometry Part B*.

[17] de Jager W, Prakken B, Rijkers GT (2009). Cytokine multiplex immunoassay: methodology and (clinical) applications. *Methods in Molecular Biology*.

[18] Badot V, Galant C, Toukap AN (2009). Gene expression profiling in the synovium identifies a predictive signature of absence of response to adalimumab therapy in rheumatoid arthritis. *Arthritis Research and Therapy*.

[19] Emery P, Nam J, Villeneuve E (2009). The role of biomarkers in the management of patients with rheumatoid arthritis. *Current Rheumatology Reports*.

[20] Deane KD, O'Donnell CI, Hueber W (2010). The number of elevated cytokines and chemokines in preclinical seropositive rheumatoid arthritis predicts time to diagnosis in an age-dependent manner. *Arthritis and Rheumatism*.

[21] Kim WU, Min SY, Cho ML (2000). The role of IL-12 in inflammatory activity of patients with rheumatoid arthritis (RA). *Clinical and Experimental Immunology*.

[22] Stabler T, Piette JC, Chevalier X, Marini-Portugal A, Kraus VB (2004). Serum cytokine profiles in relapsing polychondritis suggest monocyte/macrophage activation. *Arthritis and Rheumatism*.

[23] Hitchon CA, Alex P, Erdile LB (2004). A distinct multicytokine profile is associated with anti-cyclical citrullinated peptide antibodies in patients with early untreated inflammatory arthritis. *Journal of Rheumatology*.

[24] Alex P, Szodoray P, Knowlton N (2007). Multiplex serum cytokine monitoring as a prognostic tool in rheumatoid arthritis. *Clinical and Experimental Rheumatology*.

[25] Chen DY, Hsieh TY, Chen YM, Hsieh CW, Lan JL, Lin FJ (2009). Proinflammatory cytokine profiles of patients with elderly-onset rheumatoid arthritis: a comparison with younger-onset disease. *Gerontology*.

[26] Hong KH, Cho ML, Min SY (2007). Effect of interleukin-4 on vascular endothelial growth factor production in rheumatoid synovial fibroblasts. *Clinical and Experimental Immunology*.

[27] Watari K, Nakao S, Fotovati A (2008). Role of macrophages in inflammatory lymphangiogenesis: enhanced production of vascular endothelial growth factor C and D through NF-*κ*B activation. *Biochemical and Biophysical Research Communications*.

[28] Taylor PC (2005). Serum vascular markers and vascular imaging in assessment of rheumatoid arthritis disease activity and response to therapy. *Rheumatology*.

[29] Ozgonenel L, Cetin E, Tutun S, Tonbaklar P, Aral H, Guvenen G (2010). The relation of serum vascular endothelial growth factor level with disease duration and activity in patients with rheumatoid arthritis. *Clinical Rheumatology*.

[30] Arnett FC, Edworthy SM, Bloch DA (1988). The American Rheumatism Association 1987 revised criteria for the classification of rheumatoid arthritis. *Arthritis and Rheumatism*.

[31] Inoue E, Yamanaka H, Hara M, Tomatsu T, Kamatani N (2007). Comparison of Disease Activity Score (DAS)28- erythrocyte sedimentation rate and DAS28- C-reactive protein threshold values. *Annals of the Rheumatic Diseases*.

[32] Wells G, Becker JC, Teng J (2009). Validation of the 28-joint Disease Activity Score (DAS28) and European League Against Rheumatism response criteria based on C-reactive protein against disease progression in patients with rheumatoid arthritis, and comparison with the DAS28 based on erythrocyte sedimentation rate. *Annals of the Rheumatic Diseases*.

[33] Kirwan JR, Reeback JS (1986). Stanford Health Assessment Questionnaire modified to assess disability in British patients with rheumatoid arthritis. *British Journal of Rheumatology*.

[34] Mäkinen H, Kautiainen H, Hannonen P (2007). Disease activity score 28 as an instrument to measure disease activity in patients with early rheumatoid arthritis. *Journal of Rheumatology*.

[35] Emery P, Gabay C, Kraan M, Gomez-Reino J (2007). Evidence-based review of biologic markers as indicators of disease progression and remission in rheumatoid arthritis. *Rheumatology International*.

[36] Ibelgauft H (2010). Horst Ibelgaufts' Cope: cytokines & cells online pathfinder encyclopedia, cytokine concentrations in biological fluid.

[37] Milman N, Karsh J, Booth RA (2010). Correlation of a multi-cytokine panel with clinical disease activity in patients with rheumatoid arthritis. *Clinical Biochemistry*.

[38] Kokkonen H, Soderstrom I, Rocklov J, Hallmans G, Lejon K, Dahlqvist SR (2010). Up-regulation of cytokines and chemokines predates the onset of rheumatoid arthritis. *Arthritis and Rheumatism*.

[39] Zhu J, Paul WE (2008). CD4 T cells: fates, functions, and faults. *Blood*.

[40] Gordon S, Taylor PR (2005). Monocyte and macrophage heterogeneity. *Nature Reviews Immunology*.

[41] Mantovani A, Sica A, Locati M (2005). Macrophage polarization comes of age. *Immunity*.

[42] Kamel Mohamed SG, Sugiyama E, Shinoda K (2005). Interleukin-4 inhibits RANKL-induced expression of NFATc1 and c-Fos: a possible mechanism for downregulation of osteoclastogenesis. *Biochemical and Biophysical Research Communications*.

[43] Arpa L, Valledor AF, Lloberas J, Celada A (2009). IL-4 blocks M-CSF-dependent macrophage proliferation by inducing p21 in a STAT6-dependent way. *European Journal of Immunology*.

[44] Moroguchi A, Ishimura K, Okano K, Wakabayashi H, Maeba T, Maeta H (2004). Interleukin-10 suppresses proliferation and remodeling of extracellular matrix of cultured human skin fibroblasts. *European Surgical Research*.

[45] Groux H, Cottrez F (2003). The complex role of interleukin-10 in autoimmunity. *Journal of Autoimmunity*.

[46] Harada S, Yamamura M, Okamoto H (1999). Production of interleukin-7 and interleukin-15 by fibroblast-like synoviocytes from patients with rheumatoid arthritis. *Arthritis and Rheumatism*.

[47] Van Roon JAG, Glaudemans KAFM, Bijilsma JWJ, Lafeber FPJG (2003). Interleukin 7 stimulates tumour necrosis factor *α* and Th 1 cytokine production in joints of patients with rheumatoid arthritis. *Annals of the Rheumatic Diseases*.

[48] Hartgring SAY, Bijlsma JWJ, Lafeber FPJG, Van Roon JAG (2006). Interleukin-7 induced immunopathology in arthritis. *Annals of the Rheumatic Diseases*.

[49] Van Roon JAG, Lafeber FPJG (2008). Role of interleukin-7 in degenerative and inflammatory joint diseases. *Arthritis Research and Therapy*.

[50] Churchman SM, Ponchel F (2008). Interleukin-7 in rheumatoid arthritis. *Rheumatology*.

[51] Vandooren B, Noordenbos T, Ambarus C (2009). Absence of a classically activated macrophage cytokine signature in peripheral spondylarthritis, including psoriatic arthritis. *Arthritis and Rheumatism*.

[52] Carvalho JF, Blank M, Shoenfeld Y (2007). Vascular endothelial growth factor (VEGF) in autoimmune diseases. *Journal of Clinical Immunology*.

[53] Zendman AJW, Vossenaar ER, van Venrooij WJ (2004). Autoantibodies to citrullinated (poly)peptides: a key diagnostic and prognostic marker for rheumatoid arthritis. *Autoimmunity*.

[54] Raza K, Breese M, Nightingale P (2005). Predictive value of antibodies to cyclic citrullinated peptide in patients with very early inflammatory arthritis. *Journal of Rheumatology*.

[55] Mathsson L, Mullazehi M, Wick MC (2008). Antibodies against citrullinated vimentin in rheumatoid arthritis: higher sensitivity and extended prognostic value concerning future radiographic progression as compared with antibodies against cyclic citrullinated peptides. *Arthritis and Rheumatism*.

[56] Soós L, Szekanecz Z, Szabó Z (2007). Clinical evaluation of anti-mutated citrullinated vimentin by ELISA in rheumatoid arthritis. *Journal of Rheumatology*.

[57] Berner B, Akça D, Jung T, Muller GA, Reuss-Borst MA (2000). Analysis of Th1 and Th2 cytokines expressing CD4+ and CD8+ T cells in rlaeumatoid arthritis by flow cytometry. *Journal of Rheumatology*.

[58] Raza K, Falciani F, Curnow SJ (2005). Early rheumatoid arthritis is characterized by a distinct and transient synovial fluid cytokine profile of T cell and stromal cell origin. *Arthritis Research and Therapy*.

[59] Arend WP, Dayer JM (1990). Cytokines and cytokine inhibitors or antagonists in rheumatoid arthritis. *Arthritis and Rheumatism*.

[60] Andreakos ETH, Foxwell BMJ, Brennan FM, Feldman M, St. Clair WE, Pisetsky DS, Haynes BF (2004). Role of cytokines. *Rheumatoid Artritis*.

[61] McInnes IB, Schett G (2007). Cytokines in the pathogenesis of rheumatoid arthritis. *Nature Reviews Immunology*.

[62] Brennan FM, McInnes IB (2008). Evidence that cytokines play a role in rheumatoid arthritis. *Journal of Clinical Investigation*.

[63] van den Ham HJ, de Jager W, Bijlsma JWJ, Prakken BJ, de Boer RJ (2009). Differential cytokine profiles in juvenile idiopathic arthritis subtypes revealed by cluster analysis. *Rheumatology*.

